# 48-Week Efficacy and Safety of Dolutegravir Relative to Commonly Used Third Agents in Treatment-Naive HIV-1–Infected Patients: A Systematic Review and Network Meta-Analysis

**DOI:** 10.1371/journal.pone.0105653

**Published:** 2014-09-04

**Authors:** Dipen A. Patel, Sonya J. Snedecor, Wing Yu Tang, Lavanya Sudharshan, Jessica W. Lim, Robert Cuffe, Sonia Pulgar, Kim A. Gilchrist, Rodrigo Refoios Camejo, Jennifer Stephens, Garrett Nichols

**Affiliations:** 1 Pharmerit International, Bethesda, Maryland, United States of America; 2 GlaxoSmithKline, Stockley Park, United Kingdom; 3 ViiV Healthcare, Middlesex, United Kingdom; 4 GlaxoSmithKline, Renaissance, Pennsylvania, United States of America; 5 GlaxoSmithKline, Brentford, United Kingdom; 6 GlaxoSmithKline, Research Triangle Park, North Carolina, United States of America; University of Pittsburgh, United States of America

## Abstract

**Background:**

A network meta-analysis can provide estimates of relative efficacy for treatments not directly studied in head-to-head randomized controlled trials. We estimated the relative efficacy and safety of dolutegravir (DTG) versus third agents currently recommended by guidelines, including ritonavir-boosted atazanavir (ATV/r), ritonavir-boosted darunavir (DRV/r), efavirenz (EFV), cobicistat-boosted elvitegravir (EVG/c), ritonavir-boosted lopinavir (LPV/r), raltegravir (RAL), and rilpivirine (RPV), in treatment-naive HIV-1–infected patients.

**Methods:**

A systematic review of published literature was conducted to identify phase 3/4 randomized controlled clinical trials (up to August 2013) including at least one third agent of interest in combination with a backbone nucleoside reverse transcriptase inhibitor (NRTI) regimen. Bayesian fixed-effect network meta-analysis models adjusting for the type of nucleoside reverse transcriptase inhibitor backbone (tenofovir disoproxil fumarate/emtricitabine [TDF/FTC] or abacavir/lamivudine [ABC/3TC]) were used to evaluate week 48 efficacy (HIV-RNA suppression to <50 copies/mL and change in CD4+ cells/µL) and safety (lipid changes, adverse events, and discontinuations due to adverse events) of DTG relative to all other treatments. Sensitivity analyses assessing the impact of NRTI treatment adjustment and random-effects models were performed.

**Results:**

Thirty-one studies including 17,000 patients were combined in the analysis. Adjusting for the effect of NRTI backbone, treatment with DTG resulted in significantly higher odds of virologic suppression (HIV RNA<50 copies/mL) and increase in CD4+ cells/µL versus ATV/r, DRV/r, EFV, LPV/r, and RPV. Dolutegravir had better or equivalent changes in total cholesterol, LDL, triglycerides, and lower odds of adverse events and discontinuation due to adverse events compared to all treatments. Random-effects and unadjusted models resulted in similar conclusions.

**Conclusion:**

Three clinical trials of DTG have demonstrated comparable or superior efficacy and safety to DRV, RAL, and EFV in HIV-1–infected treatment-naive patients. This network meta-analysis suggests DTG is also favorable or comparable to other commonly used third agents (ATV/r, LPV/r, RPV, and EVG/c).

## Introduction

Two of the primary goals of anti-HIV therapy are to suppress plasma HIV viral replication and preserve and restore the number of circulating CD4+ T cells, the immune cells attacked by HIV [1], [2]. Highly active antiretroviral therapy (HAART) has achieved these goals for many patients, resulting in reduction of HIV-associated morbidity and prolonging survival to nearly that of the normal population [3], [4]. For treatment-naive patients, HAART typically includes a combination of two nucleoside reverse transcriptase inhibitors (NRTIs, the “backbone”) with one or more drugs from the more potent classes (the “third agent”) [1], [2]. The US Department of Health and Human Services (DHHS) and the European AIDS Clinical Society guidelines have recommended several third agents for the treatment of infection: ritonavir-boosted atazanavir (ATV/r), darunavir (DRV/r), lopinavir (LPV/r), efavirenz (EFV), cobicistat-boosted elvitegravir (EVG/c), raltegravir (RAL), and rilpivirine (RPV) [1], [2]. Of these, RPV and LPV/r are recommended as alternative regimen options by DHHS [2]. Many of these regimens have comparable efficacy but vary in dosing frequency, pill burden, drug interactions, and potential side effects.

Initial choice of therapy is central to long-term management of HIV infection as treatment switching has been associated with higher healthcare costs and increased likelihood of treatment failure [5]–[7]. Therefore, use of safe, well-tolerated, and effective regimens is important to allow patients to achieve long-term virologic suppression from the start of initial therapy, which may lead to improved clinical and economic outcomes including improved immune function, quality of life, and ability to control other comorbid conditions [8], [9].

Dolutegravir (DTG) has recently been approved for the treatment of HIV-1 disease in combination with other antiretroviral agents. DTG has been shown to exhibit a higher barrier to resistance compared to RAL and EVG, is dosed once daily, and has limited drug interactions including no food restrictions [10]. Three phase 3 clinical trials have shown DTG superiority to EFV [11] and DRV/r [12] and non-inferiority to RAL [13] as first-line treatment; evidence versus other guideline-recommended third agents has not yet been explored. The objective of this study is to estimate the efficacy and safety of DTG relative to other guideline-recommended agents in a Bayesian network meta-analysis (NMA). Results of this analysis will help understand comparability of DTG to all recommended agents.

## Methods

### Identification and selection of study data

The PubMed/MEDLINE, Embase, and Cochrane databases were systematically searched (up to August 2013) to identify randomized controlled trials (RCTs) evaluating efficacy and/or safety of ATV/r, DRV/r, DTG, EFV, EVG/c, LPV/r, RAL, or RPV in treatment-naive HIV-1 patients. PubMed and EMBASE search terms were *“HIV-1 [mesh] OR HIV infections [mesh] NOT pregnancy [mesh] AND ((dolutegravir OR GSK1349572) OR (efavirenz OR Sustiva OR Stocrin OR DMP-266) OR (raltegravir OR Isentress OR MK-0518) OR (elvitegravir OR GS-9137 OR JTK-303) OR (rilpivirine OR Edurant OR TMC 278) OR (darunavir OR Prezista OR TMC-114) OR (atazanavir OR Reyataz OR BMS-232632) OR (lopinavir OR ABT-378 OR Aluviran OR Koletra OR Kaletra) OR (Atripla OR Quad OR Stribild OR Eviplera OR Complera))”*. The ClinicalTrials.gov registry, US FDA summary basis of approvals, EMA EPAR scientific discussions, and references of published systematic reviews and meta-analyses were also searched for any additional data. Abstracts of the 2013 meeting of the International AIDS Society and the Interscience Conference on Antimicrobial Agents and Chemotherapy were searched to identify recent presentations. Two phase 3 studies of DTG with data available after August 2013 were also included.

Study selection was conducted by two independent researchers who performed an initial review and selection of study titles/abstracts followed by full text review and selection. Disagreements between the reviewers were resolved by consensus. Pre-specified inclusion criteria included treatment-naive patients with HIV-1 infection; studies published in English; phase 3 or 4 RCT; patients aged ≥13 years; use of at least one of the third agents of interest; and reporting at least one of the efficacy outcomes of interest after 48 weeks of treatment. Non-randomized observational studies; single-arm studies; and studies examining different dosages of the same drug, structured treatment interruptions, maintenance treatments, or treatment switching were excluded, as were publications where outcomes specific to a treatment-naive population could not be distinguished. Studies reporting outcomes such that results could not be obtained for each treatment arm individually were also excluded. The Preferred Reporting Items for Systematic Reviews and Meta-Analyses (PRISMA) guidelines were followed through all phases in the study [14].

Three researchers independently abstracted data from the final selection of studies into a structured Microsoft Access database and data were reconciled for accuracy. The Effective Public Health Practice Project Quality Assessment, a quality assessment tool, was used to assess selection bias, study design, confounders, blinding, data collection methods, and withdrawals and dropouts [15].

### Data analysis

Efficacy outcomes analyzed were virologic suppression of HIV RNA<50 copies/mL (intention-to-treat [ITT] populations, Missing/Non-Completers = Failure) and CD4+ cell change from baseline (ITT). On the basis of FDA guidance to industry [16], the following algorithms for virologic suppression were considered comparable: FDA Snapshot-50, confirmed virologic response-50, Time to Loss of Virologic Response-50, and HIV RNA<50 copies/mL. Safety outcomes analyzed were total cholesterol (TC), high-density lipoprotein (HDL), low-density lipoprotein (LDL), and triglyceride (TG) changes from baseline, adverse events (AEs; all grades due to any reason), and discontinuations due to AEs.

A Bayesian NMA framework was used to generate estimates of relative treatment outcomes [17]. This approach statistically combines the data from all clinical trials within an integrated analysis to generate a pooled estimate of the relative treatment effect of each intervention compared to all others. Models were programmed and executed using WinBUGS version 1.4.3 [18].

Treatment effects for virologic suppression, AEs, and discontinuation outcomes are estimated as odds ratios (OR) of DTG relative to a comparator. Relative CD4+ cell change and lipid changes are estimated as the mean “difference of difference” from baseline to week 48. Uncertainty around point estimates is measured by the 95% credible interval (CrI), which indicates that the outcome estimate falls within the given range with 95% probability. Credible intervals of ORs not including 1 and CrIs of mean differences not including 0 are considered “statistically significant.” Homogeneity of virologic suppression, CD4+ cell change, and discontinuation treatment effects were assessed by Q statistic (chi-square test) for pairs of third-agent treatment comparisons with three or more available studies.

Differential NRTI backbone effects independent of the third agent on treatment efficacy and lipid changes have been observed in the literature [19]–[21]. In an effort to more accurately estimate the independent effect of the third agents of interest, we included statistical adjustment for the type of NRTI backbone within the meta-analysis models (details presented in Appendix S1). Backbones were categorized into three groups: tenofovir disoproxil fumarate/emtricitabine (TDF/FTC), abacavir/lamivudine (ABC/3TC), and all other (including investigator “choice”), as no evidence was found to support distinction among other backbone regimens. Backbone regimen adjustment was possible in the analysis for virologic suppression, CD4+ change and lipids, but not AEs and discontinuations due to an insufficient number of studies and no strong clinical relevance in the case of discontinuation.

Fixed-effect models for all outcomes were chosen based on the deviance information criterion and the presence of only one study for many pairs of treatment comparisons. Limited data to estimate random-effects model parameters have been noted to lead to poor estimation of the width of the distribution of intervention effects [22]. To evaluate the robustness of the overall conclusions on the choice of model selection, backbone-unadjusted and random-effects model results are also presented (see Appendix S2 for random-effects model results).

Consistency of the modeled outcomes with observed trial data from studies not including EFV was evaluated as a measure of model validity. Results were considered consistent if the outcomes for the comparisons reported in the trials were similar to the same comparisons estimated from the model [23]. For binary outcomes, such as virologic suppression, consistency was measured by the ratio of the ORs of the direct and indirect estimates. For other continuous outcomes the difference of the mean changes from baseline between the indirect and direct estimates were calculated. If the 95% CrI for these values did not include 1 or 0 for the 2 measures, respectively, model results were considered inconsistent.

## Results

**Figure 1:**
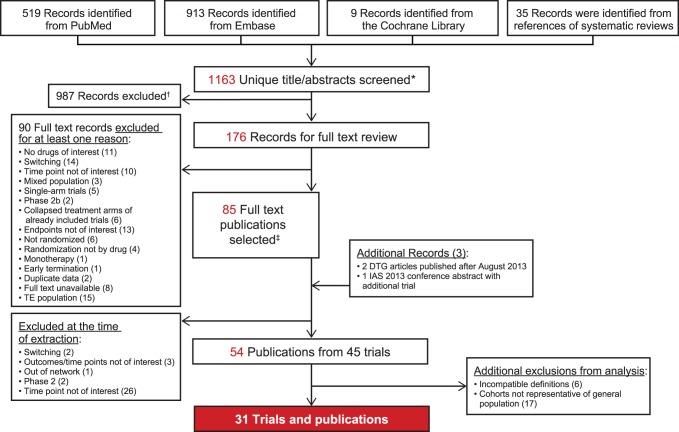
Preferred Reporting Items for Systematic Reviews and Meta-Analyses (PRISMA) Flow Chart. PubMed/MEDLINE, Embase, and Cochrane databases were searched to identify randomized controlled trials evaluating efficacy and/or safety of ATV/r, DRV/r, DTG, EFV, EVG/c, LPV/r, RAL, or RPV in treatment-naive HIV-1–infected patients. Records were screened by independent researchers, who selected study titles and abstracts for full text review. Following several rounds of exclusion based on multiple criteria, 31 trials and publications were selected for subsequent analysis. *Additional records were identified via ClinicalTrials.gov, the Food and Drug Administration (FDA), scientific discussions of the European Medicines Agency (EMA)/European Public Assessment Reports (EPAR), and third-agent package inserts. Each of these were found to be included in initial search records and noted as such. ^†^Reasons for exclusion at time of full text review: non-randomized trial; Phase 1/Phase 2 trials; patient population age <13 years; outcomes not of interest; trial duration <12 weeks; and out-of-network comparator. ^‡^34 publications were matches to ClinicalTrials.gov registry results (NCTs) to ensure comprehensive extraction of all available data pertaining to outcomes of interest.

A total of 1163 unique title/abstracts were screened from all search sources, where 176 records were selected for full text review and 54 publications representing 45 unique clinical trials were selected for data abstraction. After data abstraction, 23 articles were excluded from the meta-analysis, including 17 that did not present HIV cohorts representative of the general population [24]–[40] and 6 with incomparable virologic suppression definitions (i.e., did not define ITT/PP population and/or treatment of non-completers/missing data) [41]–[46]. Ultimately, 31 RCTs were included into the meta-analysis, representing data from 17,000 HIV-infected patients (Figure 1) with 26 reporting virologic suppression data, 28 CD4+ cell change, 20 TC, 19 HDL, 17 LDL, 17 TG, 11 AEs, and 18 studies reporting discontinuation due to AEs.

EFV was the most prevalent treatment arm included in the studies (n = 20), followed by ATV/r (n = 9), LPV/r (n = 8), DRV/r (n = 3), DTG (n = 3), RPV (n = 3), EVG/c (n = 2), and RAL (n = 2). Studies were found to be generally similar with respect to age and baseline clinical characteristics (Table S1) [11]–[13], [19], [20], [46]–[71]. The majority of patients were male (mean, 79.6%; range, 53.3%–93.1%) with mean age ranging from 29 to 40 years. Average baseline CD4+ cell count in the studies ranged from 150 to 396 cells/µL and log_10_ HIV RNA levels ranged from 4.52 to 5.41 copies/mL. All but one study included more than 50 patients per treatment arm and only 8 of the 31 included less than 100 (range of 31–465 patients). No statistically significant heterogeneity among treatment effects was identified for the EFV-RPV (*p* = 0.78; 3 studies) and EFV-LPV/r (*p* = 0.13; 3 studies) comparisons, the only comparisons associated with more than 2 studies.

**Figure 2:**
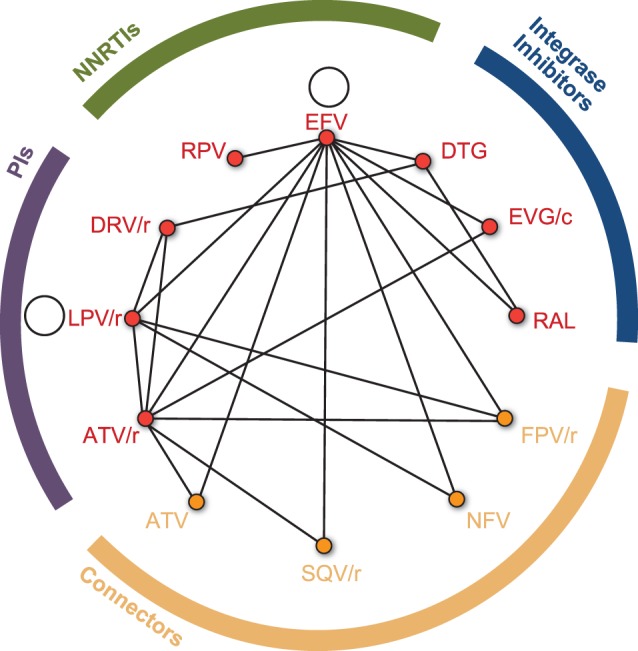
Network of treatment comparisons contained within the identified clinical trials. The major classes of third agents studied in the selected trials are indicated along the perimeter of the figure: NNRTIs, green; integrase inhibitors and PIs, purple; connectors, yellow. Black lines connecting each of the treatments of interest (red dots) represent a publication or clinical trial containing those two agents. Connector agents are drugs identified in 2 or more trials, and which were compared to 2 or more treatments of interest; connector agents are also members of the PI class. ATV = atazanavir; ATV/r = ritonavir-boosted atazanavir; DTG = dolutegravir; DRV/r = ritonavir-boosted darunavir; EFV = efavirenz; EVG/c = cobicistat-boosted elvitegravir; FPV/r = ritonavir-boosted fosamprenavir; LPV/r = lopinavir-boosted ritonavir; NFV = nelfinavir; NNRTI = non-nucleoside reverse transcriptase inhibitor; PI = protease inhibitor; RAL = raltegravir; RPV = rilpivirine; SQV/r = ritonavir-boosted saquinavir.

Figure 2 displays the network of identified treatment comparisons included in the meta-analysis. Every study did not report every outcome (Table S1), and thus networks for individual outcome analyses varied. All studies included in the analysis examined at least one third agent of interest. “Connector” third agents (ATV, saquinavir-boosted ritonavir [SQV/r], fosamprenavir-boosted ritonavir [FPV/r], and nelfinavir [NFV]) were also included when 2 or more trials were identified comparing those agents to 2 or more treatments of interest. Inclusion of such connector treatments is recommended by published guidelines [72] since it provides additional indirect evidence. Trials with treatment arms examining different backbone NRTI regimens in combination with the same third agent were included in backbone-adjusted analyses.

### Virologic suppression and CD4+ cell count change

**Figure 3:**
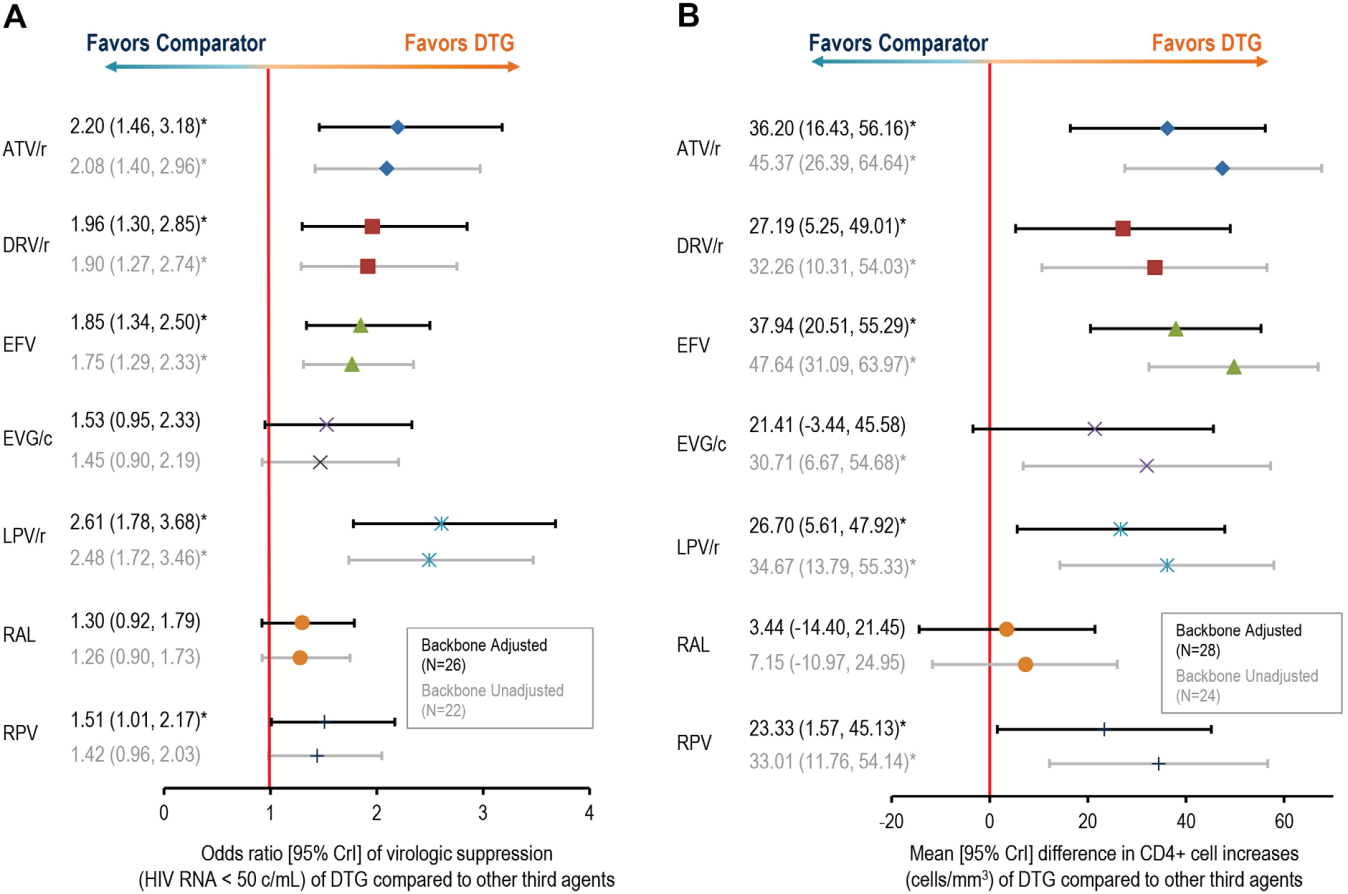
Comparison of immunologic endpoints with dolutegravir versus third agents of interest. (**A**) Odds ratio [95% CrI] for virologic suppression (HIV RNA<50 c/mL) of DTG compared with other third agents. Odds ratio values greater than 1 indicate the comparison favors DTG; CrI intervals that do not contain 1 are considered statistically significant. (**B**) Mean [95% CrI] CD4+ cell increase with dolutegravir versus third agents of interest. Crls of mean differences that do not contain 0 are considered statistically significant. ATV = atazanavir; ATV/r = ritonavir-boosted atazanavir; Crl = credible interval; DTG = dolutegravir; DRV/r = ritonavir-boosted darunavir; EFV = efavirenz; EVG/c = cobicistat-boosted elvitegravir; FPV/r = ritonavir-boosted fosamprenavir; LPV/r = lopinavir-boosted ritonavir; NFV = nelfinavir; RAL = raltegravir; RPV = rilpivirine; SQV/r = ritonavir-boosted saquinavir. *Indicates statistically significant comparison.

Mean odds of virologic suppression (HIV RNA<50 copies/mL) were significantly higher for DTG than ATV/r, DRV/r, EFV, LPV/r, and RPV (Figure 3a). Backbone-unadjusted ORs of DTG were similar but slightly lower than the adjusted model results for all comparators (which affected the significance of treatment difference versus RPV). Similar to virologic suppression, DTG was estimated to have significantly higher mean CD4+ cell increases than ATV/r, DRV/r, EFV, LPV/r, and RPV (Figure 3b). The relative increase in CD4+ count was highest for DTG compared to EFV (37.9 cells/µL [95% CrI: 20.5,55.39]). Model results that were unadjusted for the NRTI backbone generated higher mean increases for DTG relative to all comparators, which resulted in DTG gaining significance compared to EVG/c. Random-effects model results were similar (see Appendix S2). Pooled estimates of the absolute probability of achieving virologic suppression and absolute mean CD4+ changes at week 48 are shown in Table 1.

**Table 1:** Estimated probability of virologic suppression and absolute CD4+ cell count change from baseline.

	Estimated Probability of Virologic Suppression at week 48 Mean (95% CrI)				Estimated CD4^+^ cell count change from baseline to week 48 Mean (95% CrI)			
Third agent	TDF/FTC	ABC/3TC	Other	Backboneunadjusted	TDF/FTC	ABC/3TC	Other	Backboneunadjusted
	N = 26 studies	N = 26 studies	N = 26 studies	N = 22 studies	N = 28 studies	N = 28 studies	N = 28 studies	N = 24 studies
**ATV/r**	0.74 (0.68,0.79)	0.72 (0.66,0.78)	0.68 (0.62,0.74)	0.71 (0.66,0.76)	188.5 (176.5,200.6)	204.7 (191.4,218.4)	151.5 (134.2,169.0)	181.4 (171.0,191.8)
**DRV/r**	0.76 (0.68,0.83)	0.74 (0.66,0.81)	0.71 (0.62,0.78)	0.73 (0.65,0.80)	197.5 (175.4,219.4)	213.8 (191.8,235.3)	160.5 (135.8,185.1)	194.5 (173.6,215.4)
**DTG**	0.86 (0.81,0.90)	0.85 (0.80,0.88)	0.82 (0.77,0.87)	0.84 (0.79,0.87)	224.7 (205.6,243.7)	240.9 (224.3,257.7)	187.7 (166.5,209.1)	226.8 (210.7,242.6)
**EFV**	0.77 (0.74,0.79)	0.75 (0.72,0.78)	–0.72 (0.68,0.75)	0.75 (0.74,0.76)	186.8 (179.5,194.0)	203.0 (193.2,212.9)	149.7 (135.0,164.5)	179.1 (175.1,183.1)
**EVG/c**	0.80 (0.74,0.85)	0.79 (0.72,0.84)	0.76 (0.69,0.82)	0.78 (0.72,0.83)	203.3 (184.8,221.7)	219.5 (200.1,238.7)	166.3 (144.2,189.0)	196.1 (178.3,213.4)
**LPV/r**	0.70 (0.65,0.76)	0.68 (0.62,0.74)	0.65 (0.58,0.71)	0.68 (0.62,0.73)	198.0 (181.7,214.5)	214.2 (197.5,231.1)	161.0 (140.5,181.6)	192.1 (176.8,207.5)
**RAL**	0.83 (0.77,0.87)	0.81 (0.75,0.86)	0.78 (0.72,0.84)	0.80 (0.75,0.85)	221.3 (200.5,242.2)	237.5 (217.7,257.5)	184.2 (160.8,207.8)	219.6 (200.4,239.1)
**RPV**	0.80 (0.76,0.84)	0.79 (0.74,0.83)	0.76 (0.71,0.80)	0.78 (0.75,0.82)	201.4 (187.3,215.2)	217.6 (202.0,232.8)	164.3 (145.8,183.3)	193.8 (181.1,206.5)

Note: Estimates derived from this meta-analysis may differ from that of any given RCT, due to statistical aggregation of data from several trials.

### Lipid changes

**Figure 4:**
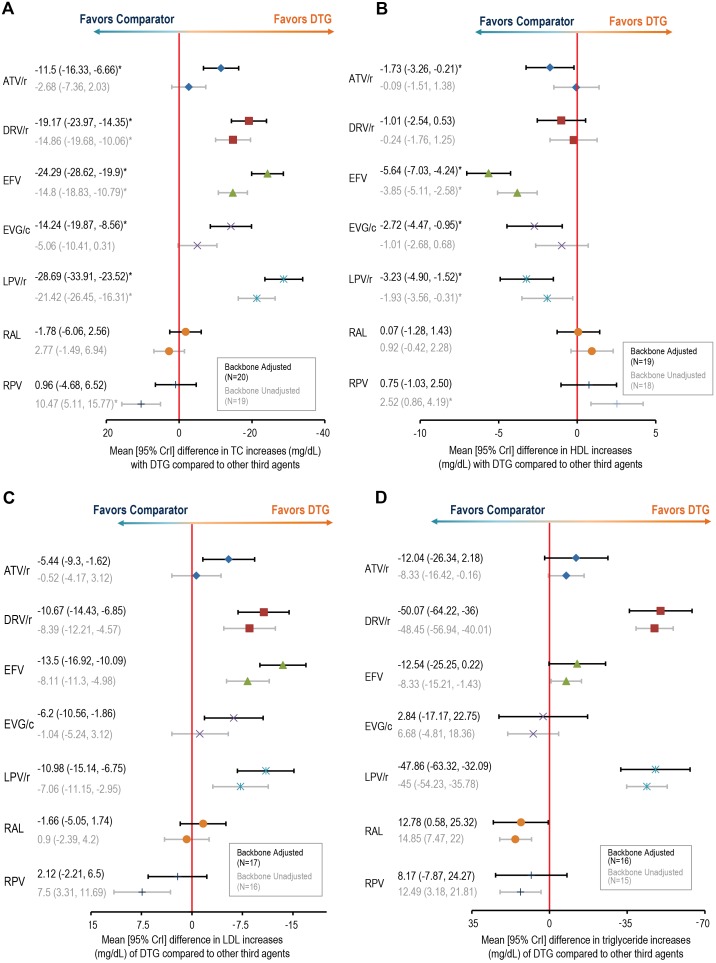
Relative changes in cholesterol and triglyceride parameters for dolutegravir versus third agents of interest. Mean changes (mg/dL [95% Crl] in lipid levels with DTG compared with other third agents are shown for (**A**) total cholesterol (TC), (**B**) HDL cholesterol, (**C**) LDL cholesterol, and (**D**) triglycerides. In all cases, Crls of mean differences that do not include 0 are considered statistically significant. ATV = atazanavir; ATV/r = ritonavir-boosted atazanavir; DTG = dolutegravir; DRV/r = ritonavir-boosted darunavir; EFV = efavirenz; EVG/c = cobicistat-boosted elvitegravir; FPV/r = ritonavir-boosted fosamprenavir; HDL = high-density lipoprotein; LDL = low-density lipoprotein; LPV/r = lopinavir-boosted ritonavir; NFV = nelfinavir; RAL = raltegravir; RPV = rilpivirine; SQV/r = ritonavir-boosted saquinavir; TC = total cholesterol. *Indicates statistically significant comparison.

DTG had significantly lower associated TC, HDL, and LDL increases (Figure 4) relative to ATV/r, DRV/r, EFV, EVG/c, and LPV/r, with the exception of DRV/r and HDL change. DTG was not significantly different than RAL or RPV in any of these lipid outcomes. Models unadjusted for the NRTI backbone resulted in slightly higher relative mean increases for DTG. Conversely, HDL changes for DTG improved, achieving insignificance rather than being significantly lower compared to ATV/r and EVG/c (and statistically improved compared with RPV).

Lower mean increases in TG were associated with DTG compared with DRV/r and LPV/r. Increases in TG were similar across all other comparisons except for RAL, for which higher mean TG increases were observed. The difference between the results for the model adjusted for NRTI versus the unadjusted model was smaller for TG than for the other lipids measured, although unadjusted results were associated with smaller uncertainty intervals, generating significantly lower and higher TG increases compared to ATV/r (−0.3 [−16.4, −0.2]) and RPV (12.5 [3.2,21.8]), respectively. Random-effects model results for lipids outcomes were similar to the fixed-effects model results (Appendix S2).

### AEs and discontinuation due to AEs

Odds of experiencing an AE were significantly lower for DTG compared to ATV/r, EFV, and LPV/r (Table 2). Odds of discontinuation due to AEs were significantly lower with dolutegravir than with all treatments except RAL and RPV. RE model results showed no significant difference in odds of AEs between DTG and any other comparator and odds of discontinuation due to AEs were lower for DTG relative to only ATV/r, EFV, and LPV/r (Appendix S2).

**Table 2:** Mean odds ratio (95% CrI) of AEs and discontinuation due to AEs.

DTG compared to	Adverse Events N = 11 studies	Discontinuation due to AEs N = 18 studies
**ATV/r**	**0.58 (0.33, 0.94)** *	**0.24 (0.10, 0.49)** *
**DRV/r**	1.06 (0.66, 1.61)	**0.45 (0.18, 0.93)** *
**EFV**	**0.57 (0.38, 0.81)** *	**0.26 (0.14, 0.43)** *
**EVG/c**	0.77 (0.41, 1.34)	**0.38 (0.15, 0.79)** *
**LPV/r**	**0.54 (0.29, 0.89)** *	**0.21 (0.09, 0.40)** *
**RAL**	1.11 (0.79, 1.53)	0.87 (0.37, 1.77)
**RPV**	0.79 (0.44, 1.30)	0.74 (0.33, 1.42)

*Significant comparisons are in bold with an asterisk.

### Model validation

Consistency was assessed for changes in degree of virologic suppression (measured by levels of HIV RNA) and levels of CD4+ T cells (cells/µL) and lipids measures by comparing modeled estimates from the network meta-analysis with the non-EFV– compared data reported directly from the studies (Table 3) [73]. Agreement was found between RCT and model estimates for all 3 measurements, with the consistency measures including 0 (for continuous CD4+ T-cell count and lipid changes) and 1 (for virologic suppression), indicating consistency between the model findings and the direct clinical trial data.

**Table 3:** Difference (95% CrI) between direct clinical trial data and indirect model estimates.

Third-agentComparisons	VirologicSuppression	CD4+	TC	HDL	LDL	TG
ATV/r v. ATV	0.85 (0.42,1.75)	17.26 (−31.72,65.88)	4.06 (−7.16,15.21)	-	-	−2.09 (−33.89,30.41)
ATV/r v. DRV/r	-	2.73 (−76.85,81.91)	2.87 (−83.98,90.72)	3.07 (−2.63,8.79)	−4.36 (−77.61,69.34)	−21.6 (−49.40,5.78)
ATV/r v. FPV/r	0.80 (0.29,2.20)	−5.39 (−72.22,62.95)	-	-	-	-
ATV/r v. LPV/r	1.08 (0.73,1.63)	6.45 (−18.75,31.74)	3.86 (−1.80,9.57)	0.50 (−1.32,2.30)	0.46 (−4.07,5.00)	8.18 (−8.63,25.25)
ATV/r v. SQV/r	1.43 (0.52,3.97)	−6.97 (−333.40,319.80)	−2.30 (−201.20,199.00)	-	-	9.63 (−190.30,209.00)
DRV/r v. LPV/r	1.03 (0.61,1.72)	3.70 (−26.68,34.16)	-	-	-	-
DTG v. DRV/r	1.05 (0.54,2.05)	0.41 (−496.50,497.90)	−0.94 (−82.29,79.6)	−0.53(−27.93,26.40)	0.37 (−63.54,63.98)	−12.62 (−191.50,167.00)
DTG v. RAL	1.04 (0.62,1.75)	3.58 (−24.46,31.33)	0.47 (−77.39,78.40)	0.16 (−29.47,29.95)	−1.02 (−62.93,60.38)	12.76 (−16.68,42.08)
EVG/c v. ATV/r	1.12 (0.63,1.99)	21.37 (−437.7,469.5)	-	-	-	-
FPV/r v. LPV/r	0.91 (0.63,1.32)	−2.38 (−29.92,25.28)	−	-	-	-
LPV/r v. NFV	-	−2.60 (−32.52,27.99)	-	-	-	-

Consistency evaluation for virologic suppression are derived OR of direct estimate divided by indirect estimates; CD4+, TC, HDL, LDL, and TG are the mean differences of direct and indirect estimates.

## Discussion

This Bayesian meta-analysis estimated efficacy and safety outcomes of DTG relative to eight first-line treatment options, providing comparative evidence to other recommended third agents that had not been assessed in randomized clinical trials. Thirty-one RCTs including 14 treatments and approximately 17,000 treatment-naive HIV-1 patients were included in the analysis. Results indicated DTG was similar to or superior to nearly all comparators of interest in every outcome. The only exceptions were: 1) HDL change, where ATV/r, EFV, EVG/c, and LPV/r demonstrated greater increases and 2) backbone-unadjusted models of TC, LDL, and TG changes, where RPV resulted in significantly lower lipid increases than DTG, though backbone-adjusted model results were not significantly different.

Results of this analysis compare to those of a smaller meta-analysis published in 2011 prior to the introduction of RPV, EVG/c, and DTG [74]. Vieira and colleagues [74] included seven studies of EFV, LPV/r, ATV/r, DRV/r, FPV/r, and RAL within a random-effects Bayesian meta-analysis to conclude that all studied treatments have similar virologic suppression efficacy at 48 weeks and that only RAL had greater improvement in CD4+ cell count at week 48 compared to EFV, which was also observed in our study. The current analysis includes data from Vieira and colleagues [74] plus 24 additional trials, which were added in part due to the inclusion of three newer third agents (8), the inclusion of connector treatments (10), and backbone adjustment, which allowed for inclusion of trials examining two arms with the same third agent (4).

Inclusion of studies of so-called connector treatments is recommended by the UK guidelines for evidence synthesis under some circumstances [72] but is not very commonly applied within NMAs, in part because NMAs are used to examine the relative outcomes of *all* relevant comparators, thus reducing the likelihood of other comparators that are not of interest. However, for the treatment of HIV, the universe of available therapies is larger than the set of guideline-recommended treatments, as newer options with greater potency, tolerability, and convenience have replaced older treatments as preferred first-line options. Although connectors were not strictly necessary in this analysis to generate a connected network, inclusion of these trials added trial data that strengthened the estimates between treatments of interest. The disadvantage of adding these treatments is the increased risk of inconsistency among the trial comparisons, but this was not observed within our model (Table 3).

We have also included statistical adjustment for the NRTI backbone regimens used in each treatment combination. This adjustment can be considered a meta-regression with the backbone category as the covariate. This feature has not been included in other published meta-analyses of HIV treatment, as most clinical trials examine two or more third agents in combination with the same NRTI backbone (or investigator choice of backbone regimen). With such trials, backbone adjustment is not necessary because NMA calculations use the relative difference between treatment arms, so the effect of the third agent independent of the NRTI backbone is the model outcome. In the case of this analysis, one study examined DTG+ABC/3TC compared to EFV+TDF/FTC. A backbone-unadjusted NMA comparison for this study would not isolate the treatment effects of DTG and EFV, necessitating the use of the NRTI backbone covariate. To provide additional information to estimate the backbone coefficients, trials comparing the same third agent with different backbones were also included. Results of these analyses indicate that backbone agents are less influential in the probability of virologic suppression, but may have a larger impact on CD4+ cell count change and lipid outcomes.

Random-effects meta-analyses tend to generate larger uncertainty intervals than fixed-effects models, which could impact conclusions of statistical significance when making comparisons among the treatments. Larger uncertainty with random-effects models was also observed in this analysis. Some comparisons with the random-effects models resulted in no significant difference between DTG and comparator where there had been significance in the fixed-effects model.

As with any scientific research, statistical significance between treatments for any clinical endpoint may not necessarily imply clinical significance of the observed effects. For virologic suppression, official guidance documents, such as the FDA guidance to industry on the development of drugs for the treatment of HIV-1 infection [16], provide explicit guidelines clinical trials must satisfy to prove non-inferiority/superiority (e.g., requiring a non-inferiority margin of 10–12 percent), and these limits can be used to imply clinical and statistical significance. However, such explicit recommendations are not available for all clinical endpoints. For CD4+ cell count, although it is predictive of disease progression [75]–[77] the clinical impact and significance of a <50 cells/mm^3^ difference in CD4+ cell recovery between two treatments (as reported in this analysis) is unknown, and has yet to be established in long-term follow-up.

DTG had lower rates of discontinuation due to adverse events compared to most of the comparators in this analysis. Integrase inhibitors have established a reputation as a class of drugs with a low rate of discontinuation that is supported by long-term follow-up results from the STARTMRK study [78]. Two of the most recently approved third agents (DTG and RPV) have shown a lower rate of discontinuations due to adverse events than their comparator EFV [11], [79], [80]. Results from this NMA align with these conclusions.

NMA methodology is subject to limitations typical to any meta-analysis as well as to some unique limitations. Notably, the results obtained represent the statistical aggregation of data from the network pool. Thus, meta-analysis results should be consistent with but are not exactly equal to any individual RCT. Results of a given meta-analysis also depend on the quality and comparability of its collection of studies. In HIV, large-scale phase 3/4 studies are generally homogeneous, and the methodologies used to conduct the included studies were consistent (Table S1). To ensure comparability of specific data inputs, only data meeting specific definitions of the virologic suppression outcome and of the algorithm for treatment of missing data were included in the analysis.

The majority of trials were similar in most study and patient characteristics, limiting any bias from potential treatment effect modifiers, such as baseline HIV RNA levels (average log_10_ HIV RNA levels ranged from 4.52–5.41 copies/mL). However, some variation existed between the studies in the average baseline CD4+ cell count, which ranged from 150 to 396 cells/µL. Hence, a secondary analysis was conducted including baseline viral load and CD4+ cell count as covariates, but no significant impact was found on the treatment effects.

Statistically significant heterogeneity was not identified for available comparisons, although it must be noted that heterogeneity tests are known to have low power to detect differences when informed by a small sample of studies [81]. Only 2 comparisons were informed by 3 trials; all remaining comparisons were based on either 1 or 2 trials. Direct and indirect RCT comparisons were available for several treatment pairs and no significant differences were found between the 2, suggesting consistency within the evidence network.

Although the scope of this analysis was limited to comparative clinical effectiveness, decision makers are increasingly using cost-effectiveness as a criterion for selection of optimal treatment strategies. Cost-effectiveness analyses of DTG have been conducted elsewhere [82], [83] and provide evidence weighing the price of DTG against its clinical advantages. To quantify these advantages relative to comparators, NMAs have become increasingly used to understand the overall clinical efficacy and safety of new treatments within the landscape of currently available options, especially when comparative RCTs including all options are impractical. The results presented herein demonstrate that the efficacy and tolerability of DTG is at least comparable to, if not better than, other recommended front-line options for the treatment of HIV-1 infection.

## Supporting Information

Table S1 **Study characteristics and outcome data.** Patient demographics; viral load, CD4+ cell count, and percent of patients with viral suppression (<50 c/mL); baseline cholesterol measurements (LDL, HDL, TC, TG); and adverse events for the trials included in this meta-analysis.
(DOC)

Appendix S1 **Model specifications.**
(DOCX)

Appendix S2 **Random-effects model results.**
(DOCX)

Checklist S1 **PRISMA Checklist.**
(DOC)
