# Supplementary material for: 48-Week Efficacy and Safety of Dolutegravir Relative to Commonly Used Third Agents in Treatment-Naive HIV-1–Infected Patients: A Systematic Review and Network Meta-Analysis
Source: PLoS One. 2014 Sep 4;9(9):e105653. doi: 10.1371/journal.pone.0105653 (PMC4154896; doi:10.1371/journal.pone.0105653)
Supplement: Table S1 — Study characteristics and outcome data. Patient demographics; viral load, CD4+ cell count, and percent of patients with viral suppression (<50 c/mL); baseline cholesterol measurements (LDL, HDL, TC, TG); and adverse events for the trials included in this meta-analysis. (DOC) [file pone.0105653.s001.doc]

**SUPPLEMENTAL MATERIALS**

**Table S1. Study characteristics and outcome data**

| **Study (Source)** | **Treatment Arms** | **N** | **% male** | **Mean age  (yrs)** | **Baseline CD4**+ **(cells/mL)** | **Baseline viral load (log10 RNA copies/mL)** | **N with virologic Suppression HIV RNA <50 copies/mL (r/n)** | **CD4+ change (cells/µL)** | **TC change (mg/dL)** | **HDL change (mg/dL)** | **LDL change (mg/dL)** | **TG change (mg/dL)** | **Adverse Events (r/n)** | **Discontinuation due to AEs (r/n)** | |
| --- | --- | --- | --- | --- | --- | --- | --- | --- | --- | --- | --- | --- | --- | --- | --- |
| 089 Study (Malan 2008) | ATV + 3TC/d4T | 95 | 72.63% | 35 | 201 | 4.8 | 71 / 95 | 174 | 24 | 9 | 22 | 14 | - | 8 / 95 | |
|  | ATV/r + 3TC/d4T | 105 | 70.48% | 34 | 194 | 5.1 | 73 / 105 | 213 | 11 | 9 | 16 | -15 | - | 1 / 105 | |
| 934 Study (Gallant 2006) | EFV + 3TC/ZDV | 254 | 87.01% | 38 | 241 | 5.0 | 173 / 254 | 158 | 35 | 9 | 20 | 31 | - | - | |
|  | EFV + TDF/FTC | 255 | 85.88% | 38 | 233 | 5.0 | 196 / 255 | 190 | 21 | 6 | 13 | 3 | - | - | |
| ACTG A5202 (Daar 2011) | ATV/r + ABC/3TC | 463 | 83.80% | 38 | 236 | 4.6 | - | 178 | 29 | 8 | 13 | 24 | - | - | |
|  | ATV/r + TDF/FTC | 465 | 83.23% | 39 | 224 | 4.7 | - | 175 | 10 | 4.8 | 2 | 14 | - | - | |
|  | EFV + ABC/3TC | 465 | 78.92% | 37 | 225 | 4.7 | - | 188 | 40 | 12 | 20 | 15 | - | - | |
|  | EFV + TDF/FTC | 464 | 84.70% | 39 | 234 | 4.7 | - | 163 | 22 | 8 | 10 | 13 | - | - | |
| ACTG A5142 (Riddler 2008) | EFV + CHOICE | 250 | 81.20% | 39 | 195 | 4.8 | 177 / 212 | - | - | - | - | - | - | - | |
|  | LPV/r + CHOICE | 253 | 76.68% | 37 | 190 | 4.8 | 165 / 217 | - | - | - | - | - | - | - | |
| ALERT (Smith 2008) | ATV/r + TDF/FTC | 53 | 88.68% | 40 | 188 | 4.9 | 44 / 53 | 183 | 10 | 14 | -6 | - | - | 1 / 53 | |
|  | FPV/r + TDF/FTC | 53 | 79.25% | 40 | 161 | 4.9 | 40 / 53 | 170 | 13 | 11 | 2 | - | - | 1 / 53 | |
| Altair (Puls 2010) | ATV/r + TDF/FTC | 105 | 71.43% | 37 | 235 | 4.8 | 93 / 105 | 192 | - | - | - | - | 95 / 105 | - | |
|  | EFV + TDF/FTC | 114 | 78.95% | 37 | 227 | 4.7 | 97 / 114 | 187 | - | - | - | - | 99 / 114 | - | |
| ARTEMIS (Mills 2009) | DRV/r + TDF/FTC | 346 | 69.65% | 35 | 218 | 4.8 | 276 / 346 | 141 | 31 | 7 | 12 | 9 | 328 / 346 | 24 / 346 | |
|  | LPV/r + TDF/FTC | 343 | 69.68% | 36 | 228 | 4.9 | 287 / 343 | 137 | 22 | 5 | 12 | 23 | 309 / 343 | 12 / 343 | |
| ASSERT (Post 2010*) | EFV + ABC/3TC | 192 | 82.81% | 38 | 240 | 5.0 | 114 / 192 | 150 | - | - | - | - | - | - | |
|  | EFV + TDF/FTC | 193 | 79.79% | 36 | 230 | 5.1 | 137 / 193 | 150 | - | - | - | - | - | - | |
| BASIC Study (Vrouenraets 2011) | ATV/r + TDF/FTC | 61 | 86.89% | 38 | 249 | 4.8 | 48 / 61 | 161 | 8.88 | 4.63 | - | 1.77 | - | - | |
|  | SQV/r + TDF/FTC | 57 | 82.46% | 39 | 234 | 4.7 | 43 / 57 | 190 | 9.65 | 8.88 | - | 1.77 | - | - | |
| CASTLE (Molina 2008) | ATV/r + TDF/FTC | 440 | 68.64% | 34 | 205 | 5.0 | 343 / 440 | 203 | 17 | 9 | 11 | 14 | 400 / 441 | 10 / 438 | |
|  | LPV/r + TDF/FTC | 443 | 68.62% | 36 | 204 | 5.0 | 338 / 443 | 219 | 38 | 11 | 17 | 58 | 399 / 437 | 14 / 440 | |
| CLASS (Bartlett 2006) | EFV + ABC/3TC | 97 | 82.47% | 37 | 307 | 4.9 | 73 / 97 | 194 | - | - | - | - | - |  | |
|  | FPV/r + ABC/3TC | 96 | 86.46% | 36 | 306 | 4.9 | 57 / 96 | 167 | - | - | - | - | - |  | |
| CNA30024 (DeJesus 2004) | EFV + 3TC/ZDV | 325 | 82.15% | 35 | 258 | 4.8 | 224 / 325 | 155 | - | - | - | - | - |  | |
|  | EFV + ABC/3TC | 324 | 79.63% | 35 | 267 | 4.8 | 226 / 324 | 209 | - | - | - | - | - |  | |
| ECHO (Molina 2011) | EFV + TDF/FTC | 344 | 79.94% | 36 | 257 | 5.0 | 281 / 344 | 182 | 24.3 | 9.27 | 11.97 | 14.16 | 317 / 344 | 27 / 344 | |
|  | RPV + TDF/FTC | 346 | 77.46% | 36 | 240 | 5.0 | 285 / 346 | 196 | 1.16 | 2.7 | -1.54 | -8.9 | 303 / 346 | 8 / 346 | |
| FLAMINGO (Clotet 2014) | DRV/r + ABC/3TC | 242 | 83.06% | 34 | 400 | 4.5 | 68 / 80 | 215 | 22.39 | 2.32 | 13.9 | 32.7 | 205 / 242 | 9 / 242 | |
|  | DRV/r + TDF/FTC |  |  |  |  |  | 132 / 162 |  |  |  |  |  |  |  |  |
|  | DTG + ABC/3TC | 242 | 87.19% | 34 | 390 | 4.5 | 71 / 79 | 244 | 4.25 | 1.93 | 3.09 | -5.31 | 206 / 242 | 3 / 242 | |
|  | DTG + TDF/FTC |  |  |  |  |  | 146 / 163 |  |  |  |  |  |  |  |  |
| GS-236-0102 (Sax 2012) | EFV + TDF/FTC | 352 | 89.77% | 38 | 382 | 4.8 | 296 / 352 | 206 | 18.9 | 7.72 | 16.99 | - | 334 / 352 | 18 / 352 | |
|  | EVG/c + TDF/FTC | 348 | 88.22% | 38 | 391 | 4.7 | 305 / 348 | 239 | 9.65 | 5.02 | 10.04 | - | 650 / 701** | 12 / 348 | |
| GS-236-0103 (DeJesus 2012) | EVG/c + TDF/FTC | 353 | 91.78% | 38 | 351 | 4.8 | 316 / 353 | 207 | 10 | 5.79 | 10.81 | 7.97 |  | 13 / 353 | |
|  | ATV/r + TDF/FTC | 355 | 89.01% | 39 | 366 | 4.8 | 308 / 355 | 211 | 8.11 | 5.02 | 10.42 | 23.01 | 333 / 355 | 18 / 355 | |
| HEAT (Smith 2009) | LPV/r + ABC/3TC | 343 | 83.97% | 38 | 214 | 4.9 | 216 / 343 | 201 | - | - | - | - | - | - | |
|  | LPV/r + TDF/FTC | 345 | 80.00% | 38 | 193 | 4.8 | 210 / 345 | 173 | - | - | - | - | - | - | |
| INITIO Trial (INITIO 2006) | EFV + d4T/ddI | 297 | 78.11% | 39 | 221 | 4.9 | - | 160 | - | - | - | - | - | - | |
|  | NFV + d4T/ddI | 311 | 79.42% | 39 | 223 | 5.0 | - | 162 | - | - | - | - | - | - | |
| Jemsek 2006 (Jemsek 2006) | ATV + CHOICE | 111 | 73.87% | 30 | 328 | 4.8 | - | - | 0 | 3 | -2 | -12 | - | - | |
|  | EFV + CHOICE | 100 | 71.00% | 29 | 323 | 4.7 | - | - | 31 | 7 | 17 | 15 | - | - | |
| KLEAN (Eron 2006) | FPV/r + ABC/3TC | 444 | 78.38% | 37 | 194 | 5.1 | 288 / 444 | 191 | 53 | 14 | 23 | - | - | 24 / 444 | |
|  | LPV/r + ABC/3TC | 434 | 77.88% | 38 | 188 | 5.1 | 285 / 434 | 176 | 61 | 13 | 28 | - | - | 23 / 434 | |
| Lake Study (Echeverria 2010) | EFV + ABC/3TC | 63 | 85.71% | 39 | 193 | 5.4 | 36 / 63 | 298 | 48 | - | - | - | - | 14 / 63 | |
|  | LPV/r + ABC/3TC | 63 | 87.30% | 37 | 191 | 5.3 | 40 / 63 | 249 | 44 | - | - | - | - | 8 / 63 | |
| M98-863 (Walmsley 2002) | LPV/r + 3TC/d4T | 326 | 79.75% | 38 | 260 | 4.9 | - | 207 | - | - | - | - | - | - | |
|  | NFV + 3TC/d4T | 327 | 80.73% | 37 | 258 | 4.9 | - | 195 | - | - | - | - | - | - | |
| METABOLIK (Aberg 2012) | ATV/r + TDF/FTC | 31 | 87.10% | 35 | 316 | 4.6 | - | 205 | 11.8 | 3.7 | 13.9 | 9.6 | 29 / 31 | 2 / 31 | |
|  | DRV/r + TDF/FTC | 34 | 85.29% | 37 | 267 | 5.0 | - | 217 | 22.3 | 6 | 14.7 | 26.1 | 31 / 34 | 0 / 34 | |
| Montaner 2006 (Montaner 2006) | EFV + CHOICE | 77 | 75.32% | 37 | 343 | 4.7 | 55 / 77 | 204 | 42 | 12.1 | - | 71.4 | - | - | |
|  | SQV/r + CHOICE | 75 | 70.67% | 37 | 372 | 4.8 | 38 / 75 | 239 | 32 | 8.7 | - | 51.2 | - | - | |
| NORTHIV (Andersson 2013) | ATV/r + CHOICE | 81 | 61.73% | 39 | 170 | 5.2 | 56 / 81 | - | - | - | - | - | - | 6 / 81 | |
|  | EFV + CHOICE | 77 | 53.25% | 37 | 150 | 5.3 | 62 / 77 | - | - | - | - | - | - | 5 / 77 | |
|  | LPV/r + CHOICE | 81 | 55.55% | 37 | 150 |  | 54/81 | - | - | - | - | - | - | 12 / 81 | |
| SINGLE (Walmsley 2013) | DTG + ABC/3TC | 414 | 83.82% | 36 | 334.5 | 4.7 | 364 / 414 | 267 | 17 | 5.22 | 8.49 | 17.7 | 369 / 414 | 10 / 414 | |
|  | EFV + TDF/FTC | 419 | 84.96% | 35 | 339 | 4.7 | 338 / 419 | 208 | 23.9 | 7.95 | 13.13 | 18.61 | 387 / 419 | 42 / 419 | |
| SPRING 2 (Raffi 2013) | DTG + ABC/3TC | 411 | 84.67% | 37 | 359 | 4.5 | 145 / 169 | 230 | 6.95 | 2.7 | 3.09 | 8.85 | 339 / 411 | 8 / 411 | |
|  | DTG + TDF/FTC |  |  |  |  |  | 216 / 242 |  |  |  |  |  |  |  |  |
|  | RAL + ABC/3TC | 411 | 86.37% | 35 | 362 | 4.6 | 142 / 164 | 230 | 8.88 | 2.7 | 3.47 | 8.85 | 340 / 411 | 6 / 411 | |
|  | RAL + TDF/FTC |  |  |  |  |  | 209 / 247 |  |  |  |  |  |  |  |  |
| Squires 2004 (Squires 2004) | ATV + 3TC/ZDV | 404 | 63.61% | 33 | 286 | 4.9 | 131 / 404 | 176 | - | - | - | - | - | 26 / 404 | |
|  | EFV + 3TC/ZDV | 401 | 66.08% | 33 | 280 | 4.9 | 150 / 401 | 160 | - | - | - | - | - | 34 / 401 | |
| STaR (Cohen 2013) | EFV + TDF/FTC | 392 | 93.11% | 35 | 385 | 4.8 | 320 / 392 | 191 | - | - | - | - | - | 34 / 392 | |
|  | RPV + TDF/FTC | 394 | 92.89% | 37 | 396 | 4.8 | 338 / 394 | 200 | - | - | - | - | - | 10 / 394 | |
| STARMRK (Lennox 2009) | EFV + TDF/FTC | 282 | 81.91% | 37 | 217.4 | 5.0 | 230 / 281 | 163 | 32.9 | 10.1 | 16.24 | 37.2 | 272 / 282 | 17 / 282 | |
|  | RAL + TDF/FTC | 281 | 80.78% | 38 | 218.9 | 5.0 | 241 / 280 | 189 | 10.1 | 4.25 | 5.8 | -2.7 | 253 / 281 | 8 / 281 | |
| THRIVE (Cohen 2011) | EFV + CHOICE | 338 | 72.19% | 36 | 263 | 5.0 | 276 / 338 | 171 | 30.5 | 10.4 | 16.99 | 12.39 | 312 / 338 | 25 / 338 | |
|  | RPV + CHOICE | 340 | 73.53% | 36 | 263 | 5.0 | 291 / 340 | 189 | 3.09 | 4.25 | -0.77 | -6.2 | 313 / 340 | 15 / 340 | |
| *ASSERT reported lipid values, though TC, LDL, and TG appeared to be mmol/L in magnitude rather than the reported mg/dL units. An attempt was made to contact the authors, but no response was received. As such, the study was excluded from lipid analysis; ** For AE analysis, data from these studies were combined. | | | | | | | | | | | | | | |  |
