# Supplementary material for: 48-Week Efficacy and Safety of Dolutegravir Relative to Commonly Used Third Agents in Treatment-Naive HIV-1–Infected Patients: A Systematic Review and Network Meta-Analysis
Source: PLoS One. 2014 Sep 4;9(9):e105653. doi: 10.1371/journal.pone.0105653 (PMC4154896; doi:10.1371/journal.pone.0105653)
Supplement: Appendix S1 — Model specifications. (DOCX) [file pone.0105653.s002.docx]

**Appendix S1 Model specifications**

*Binary outcome data*

Binary measures such as virologic suppression can be summarized as the number of successes $r_{i}^{a_{k},b_{k}}$ out of the total number of patients $n_{i}^{a_{k},b_{k}}$ for third agent *a* and backbone *b* of treatment arm *k* in study *i*. These data provide information on the probability of the outcome,$p_{i}^{a_{k},b_{k}}$, for each treatment arm, *k*, in study *i* as follows:

Each study has a “baseline” intervention arm, *k* = 1, with study-specific baseline log-odds of outcome, $\mu_{i}$. In adjusted analyses, the log-odds ratio of the outcome for intervention *k* ≠ 1 is divided into two parts, $\delta_{i}^{k}$ and $\beta_{i}^{k}$.

In the adjusted models, $\delta_{i}^{k}$ is the log-odds ratio of outcome for the third agent in treatment arm *k* relative to the third agent in treatment arm 1 (assuming identical backbones) and $\beta_{i}^{k}$ is the log-odds ratio of the backbone of treatment arm *k* relative to the backbone in treatment arm 1 (assuming identical third agents). In the unadjusted models, $\beta_{i}^{k}=0$ for all *k*.

The unadjusted model can be written as:

$$\text{log-odds}(p_{i}^{a_{k},b_{k}})=\left\{\begin{matrix} \mu_{i} & k=1 \\ \mu_{i}+\delta_{i}^{k}+\beta_{i}^{k} & k\neq1 \end{matrix}\right.$$

where

$\delta_{i}^{k}\sim Normal\left(d_{a_{k}}-d_{a_{1}},\tau_{a}^{2}\right)$ and $\beta_{i}^{k}\sim Normal\left(d'_{b_{k}}-d'_{b_{1}},\tau_{b}^{2}\right)$.

The fixed-effect model specification is identical except

$\delta_{i}^{k}=d_{a_{k}}-d_{a_{1}}$ and $\beta_{i}^{k}=d'_{b_{k}}-d'_{b_{1}}$.

The parameters $d_{a}$ and $d'_{b}$ represent the overall mean log-odds ratio of the outcome for each third agent *a* and each backbone *b* relative to the usual care, which are assumed to be EFV and TDF/FTC, respectively (i.e., $d_{EFV}=0$ and $d_{TDF/FTC}=0$).

*Continuous outcome data:*

Mean CD4^+^ cell change and lipid changes from baseline to week 48 were modeled as continuous outcomes. These are summarized as the mean change, $\Delta_{i}^{a_{k},b_{k}}$, for each treatment arm *k* in each study *i*, and are assumed to be normally distributed with corresponding standard error $\sigma_{i}^{a_{k},b_{k}}$. It was necessary to impute the standard error for some trials using the method presented by Stevens.[[1]](#footnote-1)

The fixed- and random-effects models for the backbone-adjusted treatment effects are identical to the binary models described above, except that the outcome measure is not transformed:

1. Stevens JW (2011). A note on dealing with missing standard errors in meta-analyses of continuous outcome measures in WinBUGS. Pharmaceut Statist 10: 374–378. [↑](#footnote-ref-1)
