# Supplementary material for: 48-Week Efficacy and Safety of Dolutegravir Relative to Commonly Used Third Agents in Treatment-Naive HIV-1–Infected Patients: A Systematic Review and Network Meta-Analysis
Source: PLoS One. 2014 Sep 4;9(9):e105653. doi: 10.1371/journal.pone.0105653 (PMC4154896; doi:10.1371/journal.pone.0105653)
Supplement: Appendix S2 — Random-effects model results. (DOCX) [file pone.0105653.s003.docx]

**Appendix S2 Random-effects model results**

Table A1. Odds ratios and risk differences of virologic suppression (random-effects models)

|  | **OR (95% CrI)** | |
| --- | --- | --- |
| **DTG** compared to | Backbone adjusted [n=26 studies] | Backbone unadjusted [n=22 studies] |
| **ATV/r** | **2.17 (1.35, 3.30)*** | **2.06 (1.28, 3.10)*** |
| **DRV/r** | **1.95 (1.20, 2.95)*** | **1.89 (1.19, 2.89)*** |
| **EFV** | **1.84 (1.26, 2.61)*** | **1.74 (1.21, 2.43)*** |
| **EVG/c** | 1.53 (0.86, 2.49) | 1.45 (0.83, 2.32) |
| **LPV/r** | **2.59 (1.67, 3.86)*** | **2.46 (1.59, 3.62)*** |
| **RAL** | 1.29 (0.87, 1.87) | 1.27 (0.84, 1.84) |
| **RPV** | 1.50 (0.93, 2.31) | 1.42 (0.88, 2.18) |

*Using reference backbone tenofovir disoproxil fumarate/emtricitabine. ATV/r=ritonavir-boosted atazanavir; DTG=dolutegravir; DRV/r=ritonavir-boosted darunavir; EFV=efavirenz; EVG/c=cobicistat-boosted elvitegravir; LPV/r=ritonavir-boosted lopinavir; RAL=raltegravir; RPV=rilpivirine.

Table A2. Mean CD4 difference from baseline (random-effects models)

|  | **Relative CD4 Difference (95% CrI)** | |
| --- | --- | --- |
| **DTG** compared to | Backbone adjusted [n=28 studies] | Backbone unadjusted [n=24 studies] |
| **ATV/r** | **36.05 (12.6, 60.39)*** | **44.63 (18.86, 69.96)*** |
| **DRV/r** | **27.19 (0.93, 53)*** | **32.17 (4.98, 58.94)*** |
| **EFV** | **37.7 (17.1, 59.68)*** | **46.08 (23.48, 67.34)*** |
| **EVG/c** | 21.17 (-7.68, 51.01) | 29.66 (-2.8, 61.44) |
| **LPV/r** | **27.46 (3.26, 52.61)*** | **35.56 (8.77, 63.27)*** |
| **RAL** | 3.59 (-17.5, 24.6) | 7.35 (-16.48, 32.4) |
| **RPV** | 23.14 (-2.92, 48.39) | **31.42 (2.52, 59.35)*** |

*Using reference backbone tenofovir disoproxil fumarate/emtricitabine. ATV/r=ritonavir-boosted atazanavir; DTG=dolutegravir; DRV/r=ritonavir-boosted darunavir; EFV=efavirenz; EVG/c=cobicistat-boosted elvitegravir; LPV/r=ritonavir-boosted lopinavir; RAL=raltegravir; RPV=rilpivirine.

Table A3. Mean total cholesterol difference from baseline (random-effects models)

|  | **Relative TC Difference (95% CrI)** | |
| --- | --- | --- |
| **DTG** compared to | Backbone adjusted* [n=20 studies] | Backbone unadjusted [n=19 studies] |
| **ATV/r** | **-11.87 (-20.5, -3.5)*** | -3.59 (-22.39, 15.18) |
| **DRV/r** | **-18.53 (-26.34, -10.04)*** | -13.4 (-32.08, 5.11) |
| **EFV** | **-24.78 (-32.74, -17.30)*** | -15.65 (-32.40, 1.22) |
| **EVG/c** | **-14.64 (-25.11, -4.61)*** | -6.06 (-29.86, 17.55) |
| **LPV/r** | **-27.04 (-35.53, -17.05)*** | -17.24 (-37.86, 3.71) |
| **RAL** | -1.97 (-10.16, 5.73) | 2.43 (-16.29, 20.81) |
| **RPV** | 0.42 (-10.07, 10.57) | 9.41 (-14.56, 33) |

*Using reference backbone tenofovir disoproxil fumarate/emtricitabine. ATV/r=ritonavir-boosted atazanavir; DTG=dolutegravir; DRV/r=ritonavir-boosted darunavir; EFV=efavirenz; EVG/c=cobicistat-boosted elvitegravir; LPV/r=ritonavir-boosted lopinavir; RAL=raltegravir; RPV=rilpivirine.

Table A4. Mean HDL difference from baseline (random-effects models)

|  | **Relative HDL Difference (95% CrI)** | |
| --- | --- | --- |
| **DTG** compared to | Backbone adjusted* [n=19 studies] | Backbone unadjusted [n=18 studies] |
| **ATV/r** | -1.67 (-3.47, 0.19) | 0.15 (-3.68, 4.06) |
| **DRV/r** | -0.96 (-2.83, 0.94) | -0.29 (-4.3, 3.67) |
| **EFV** | **-5.65 (-7.28, -3.97)*** | **-3.83 (-7.32, -0.35)*** |
| **EVG/c** | **-2.72 (-4.9, -0.46)*** | -0.87 (-5.7, 4.06) |
| **LPV/r** | **-3.08 (-5.12, -0.94)*** | -1.42 (-5.99, 3.31) |
| **RAL** | 0.08 (-1.59, 1.81) | 0.96 (-2.87, 4.76) |
| **RPV** | 0.73 (-1.48, 2.94) | 2.56 (-2.35, 7.44) |

*Using reference backbone tenofovir disoproxil fumarate/emtricitabine. ATV/r=ritonavir-boosted atazanavir; DTG=dolutegravir; DRV/r=ritonavir-boosted darunavir; EFV=efavirenz; EVG/c=cobicistat-boosted elvitegravir; LPV/r=ritonavir-boosted lopinavir; RAL=raltegravir; RPV=rilpivirine.

Table A5. Mean LDL difference from baseline (random effects)

|  | **Relative LDL Difference (95% CrI)** | |
| --- | --- | --- |
| **DTG** compared to | Backbone adjusted* [n=17 studies] | Backbone unadjusted [n=16 studies] |
| **ATV/r** | **-5.54 (-10.39, -0.85)*** | -1.47 (-13, 10.06) |
| **DRV/r** | **-10.67 (-15.3, -5.87)*** | -7.58 (-18.97, 4.27) |
| **EFV** | **-13.58 (-17.94, -9.47)*** | -8.83 (-19.22, 1.33) |
| **EVG/c** | **-6.28 (-11.86, -0.85)*** | -1.91 (-16.59, 12.68) |
| **LPV/r** | **-11.02 (-16.13, -5.62)*** | -7.03 (-20.76, 6.56) |
| **RAL** | -1.73 (-6.1, 2.57) | 0.59 (-10.89, 12.07) |
| **RPV** | 2.04 (-3.65, 7.45) | 6.82 (-7.3, 21.23) |

*Using reference backbone tenofovir disoproxil fumarate/emtricitabine. ATV/r=ritonavir-boosted atazanavir; DTG=dolutegravir; DRV/r=ritonavir-boosted darunavir; EFV=efavirenz; EVG/c=cobicistat-boosted elvitegravir; LPV/r=ritonavir-boosted lopinavir; RAL=raltegravir; RPV=rilpivirine.

Table A6. Mean triglyceride difference from baseline (random-effects models)

|  | **Relative Trig Difference (95% CrI)** | |
| --- | --- | --- |
| **DTG** compared to | Backbone adjusted* [n=16 studies] | Backbone unadjusted [n=15 studies] |
| **ATV/r** | -13.28 (-42.51, 14.49) | -10.09 (-35.54, 15.06) |
| **DRV/r** | **-46.26 (-72.44, -17.36)*** | **-42.42 (-67.44, -15.7)*** |
| **EFV** | -13.97 (-40.27, 10.64) | -10.47 (-32.89, 11.28) |
| **EVG/c** | 1.19 (-41.75, 41.54) | 4.6 (-34.97, 43.39) |
| **LPV/r** | **-45.11 (-76.93, -11.54)*** | **-41.22 (-72.06, -9.34)*** |
| **RAL** | 12.31 (-14.45, 37.86) | 14.28 (-10.66, 38.94) |
| **RPV** | 6.57 (-28.84, 39.52) | 10.07 (-21.31, 40.68) |

*Using reference backbone tenofovir disoproxil fumarate/emtricitabine. ATV/r=ritonavir-boosted atazanavir; DTG=dolutegravir; DRV/r=ritonavir-boosted darunavir; EFV=efavirenz; EVG/c=cobicistat-boosted elvitegravir; LPV/r=ritonavir-boosted lopinavir; RAL=raltegravir; RPV=rilpivirine.

Table A7. Odd ratios of AEs and discontinuation due to AEs (random-effects models)

| **DTG** compared to | **OR (AEs)** [n=11 studies] | | **OR (Discontinued due to AEs)** [n=16 studies] | |
| --- | --- | --- | --- | --- |
|  | Mean | 95% CrI | Mean | 95% CrI |
| **ATV/r** | 0.62 | (0.22, 1.31) | **0.25** | **(0.05, 0.69)*** |
| **DRV/r** | 1.11 | (0.44, 2.22) | 0.53 | (0.12, 1.60) |
| **EFV** | 0.60 | (0.27, 1.12) | **0.29** | **(0.09, 0.70)*** |
| **EVG/c** | 0.87 | (0.24, 2.11) | 0.44 | (0.07, 1.39) |
| **LPV/r** | 0.59 | (0.18, 1.38) | **0.22** | **(0.05, 0.61)*** |
| **RAL** | 1.27 | (0.60, 2.63) | 1.01 | (0.25, 2.78) |
| **RPV** | 0.87 | (0.27, 1.94) | 0.89 | (0.19, 2.65) |

*Using reference backbone tenofovir disoproxil fumarate/emtricitabine. ATV/r=ritonavir-boosted atazanavir; DTG=dolutegravir; DRV/r=ritonavir-boosted darunavir; EFV=efavirenz; EVG/c=cobicistat-boosted elvitegravir; LPV/r=ritonavir-boosted lopinavir; RAL=raltegravir; RPV=rilpivirine.
